# Novel truxene-based dipyrromethanes (DPMs): synthesis, spectroscopic characterization and photophysical properties

**DOI:** 10.3762/bjoc.20.186

**Published:** 2024-08-29

**Authors:** Shakeel Alvi, Rashid Ali

**Affiliations:** 1 Department of Chemistry, Jamia Millia Islamia, Jamia Nagar, Okhla, New Delhi-110025, India

**Keywords:** condensation, co-timerization, dipyrromethane, Friedel–Crafts acylation, heterocycles, pyrrole, truxene

## Abstract

For the first time, herein, we report the synthetic part of the truxene-centred mono-, di- and tri-substituted dipyromethanes (DPMs) in good yields (60–80%) along with their preliminary photophysical (absorption, emission and time resolved fluorescence lifetime) properties. The condensation reaction for assembling the required DPMs were catalyzed with trifluoroacetic acid (TFA) at 0 °C to room temperature (rt), and the stable dipyrromethanes were purified through silica-gel column chromatography. After successfully synthesizing these easy-to-make yet interesting molecules, they were fully characterized by means of the standard spectroscopic techniques (^1^H NMR, ^13^C NMR and HRMS). We are of the opinion that these truxene-based systems will be useful for diverse applications in future studies.

## Introduction

The scaffold of truxene (10,15‐dihydro‐5*H*‐diindeno[1,2‐*a*;1′,2′‐*c*]fluorene) and its congeners comprises three fluorene subunits – sharing a common benzene ring at the centre [1]. The notable structural signatures of truxene are its rigid, planar and *C*_3_-symmetric skeleton, wherein three peripheral phenylene ring systems are all *meta*-positioned with respect to the congested central benzene ring, so that all four benzene rings are co-planar having π‐conjugation [2–4]. Remarkably, these unique characteristics of the truxene scaffold, results in strong π–π stacking ability in addition to the strong electron‐donating capability – hinting for the truxene’s capability as a worthy building block in the advancement of cutting-edge functional materials for diverse uses [1,5–7]. Notably, to synthesize this vital heptacyclic star‐shaped π‐conjugated polyarene framework, only a single acid-mediated co-trimerization step is required from an inexpensive and commercially available starting material, namely 1-indanone [8].

It is to be pointed out, though for the first time truxene was reported in 1894 by Kipping [9], whereby 3‐phenylpropionic acid in situ cyclized under acidic conditions to indan‐1‐one which under the same conditions offered a mixture of both isomers, that is truxene as well as isotruxene. However, the practical synthesis of only truxene was established by Dehmlow’s research group in 1997 [10].

Remarkably, one of the advantages of truxene over the other polyaromatic hydrocarbons (PAHs) is the presence of three benzylic positions, that generally permit to assemble a myriad of functionalized truxene-based architectures of particular interest including the worthy bowl-shaped molecules [11]. To date a plethora of truxene and related compounds have successfully been synthesized and reported by various research groups across the world, and their diverse potential applications have also been successfully revealed [1,12–13]. The most promising applications of truxene-based systems have been found in organic photovoltaics (OPVs), dye‐sensitized solar cells (DSSCs), fluorescent probes, organic thin‐film transistors (OTFTs), lasers, organic light emitting diodes (OLEDs), liquid crystals, non-linear optical (NLO), organogels, molecular wires, self-assembly and so forth [14–25].

Moreover, nowadays these invaluable compounds have also received great attention of supramolecular chemists, and finds applications in sensing, catalysis, donor–acceptor systems, energy transfer and electron transfer processes etc. [26–28]. On the other front, doping with heteroatom(s) to the truxene skeleton drastically modulate its unique physical as well as chemical properties besides the geometrical structure, as well [29]. After successful construction of truxene and its asymmetrical isomer, that is the isotruxene scaffold [30] – having differences in clipping of fluorene moieties, chemists began to synthesize the heteroatom-doped truxenes as well as isotruxene molecules, so-called “hetero-truxenes/isotruxenes” [31–33]. As can be inspected from the scientific literature, to date a plethora of hetero-analogues of both truxene and isotruxene have been reported with altered physiochemical properties [30,34–35].

**Figure 1:**
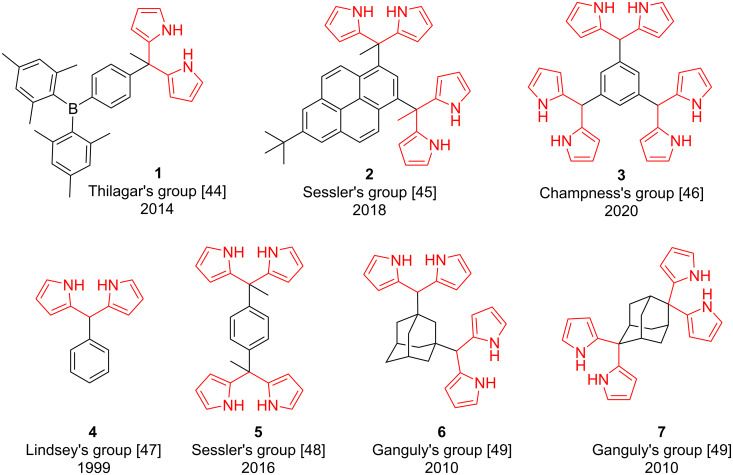
Structures of some reported mono-, di- and tri-dipyrromethane derivatives [44–49].

To our best knowledge, derivatizations of the truxene core with heterocycles are limited [33,36–38] and needs to be explored for diverse promising applications. Keeping the importance of DPMs in mind due to their utmost significance as a building block in the construction of porphyrinogens, related polypyrrolic macrocycles, and pigments [39–41]. As shown in Figure 1, these DPMs and many more have fruitfully been used by several research groups in sensing/binding of a variety of biologically important anions due to the presence of two pyrrolic NH hydrogen bond donors [38–43]. Notably, in the past few decades, the chemistry of DPMs have attested to be imperative in the existing chemical research because of their easy syntheses, good stability in addition to the stimulating photophysical properties and distinct architectures emerging from the self-assembly processes [42]. Noticeably, most extensively used DPMs belong to the 4,4-difluoro-4-bora-3a,4a-diaza-s-indacene (BODIPY), owing to their high propensity toward chemical manipulations and outstanding optical properties [43].

Herein, we present for the first time three new mono-, di-, tri-dipyrromethane appended truxene derivatives with the intention to explore them for future sensing and/or binding properties.

## Results and Discussion

**Scheme 1:**
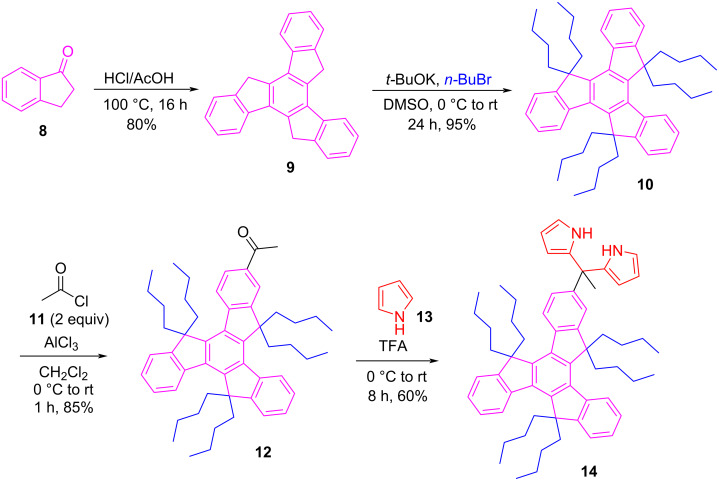
Synthesis of the mono**-**DPM-based truxene derivative **14**.

To achieve our goal towards the construction of truxene-centered DPMs (**14**, **16** and **18**), we have first prepared the truxene scaffold **9** from the inexpensive and commercially available 1-indanone (**8**) using an already reported protocol, as illustrated in Scheme 1 [38]. Moreover, the hexabutylated truxene (HexBT) framework **10**, soluble in common organic solvents, was also assembled through a literature reported method [38,50]. The reason for accomplishing the butylation was to get good solubility of the system, as the pristine truxene scaffold is insoluble in most of the commonly used organic solvents.

**Scheme 2:**
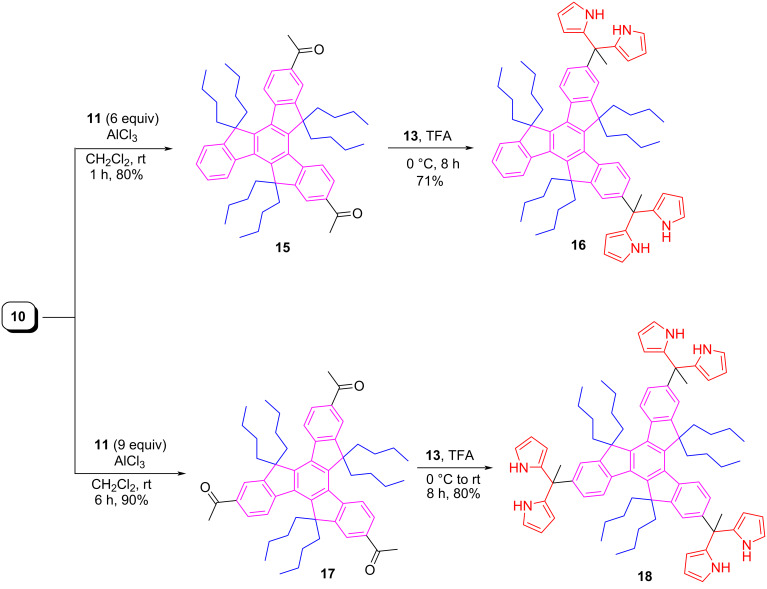
Synthesis of di- and tri-DPM-based truxene derivatives **16** and **18**.

Next, we prepared mono-, di- and triacylated truxene derivatives (**12**, **15**, and **17**) in controlled manner (with appropriate equivalents of acetyl chloride and aluminium chloride) using one-to-threefold Friedel–Crafts acylation reaction(s) at 0 °C to rt in dichloromethane (DCM) solvent (Scheme 1 and Scheme 2). Subsequent condensation of thus prepared acetylated truxenes with freshly distilled pyrrole using trifluoroacetic acid (TFA) as an acidic catalyst afforded the anticipated DPM-appended truxene derivatives (**14**, **16** and **18**) in good yields (60–80%). All the newly prepared DPM-linked truxene-hybrid molecules as well as the intermediate acetylated truxene derivatives were successfully characterized and their structures were established by means of the ^1^H and ^13^C NMR spectroscopy, besides further confirmation by mass spectrometry (see Supporting Information File 1).

### The UV–vis absorption, emission and time-resolved fluorescence spectra

**Figure 2:**
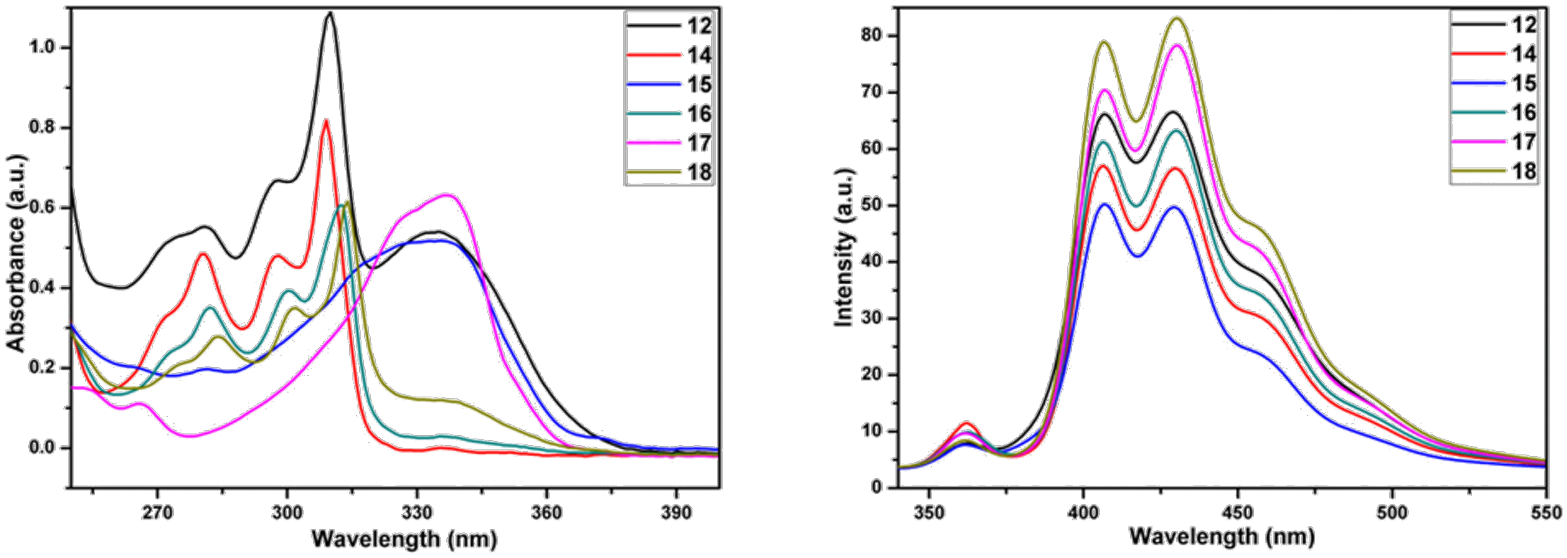
UV–vis absorption (left) and fluorescence spectra (right) recorded in chloroform.

Emission and absorption spectra of thus synthesized truxenes (**12**, **14**, **15**, **16**, **17**, and **18**) were analyzed in CHCl_3_ (Figure 2). The UV–vis spectrum of mono-acetyltruxene **12** displayed a broad band centered at 335.21 nm, an intense peak near 309.75 nm having a shoulder at 297.70 nm, and a less intense, broader band around 280.29 nm. A strong band with absorption maxima at 308.94 nm besides two more bands at ca*.* 280.29 nm, and 297.44 nm were observed for truxene-based mono-DPM **14**. On the other hand, a very broad band for example at 334.41 nm was observed in the case of diacetyltruxene derivative **15**. Moreover, for the same compound **15**, a very tiny band was also noticed at ca. 281.65 nm. In triacetylated truxene **17** two bands at 264.25 nm (less intense) and 338.68 nm (a broad and more intense) were found.

Similarly, in the di-DPM appended truxene system **16**, two bands at ca. 265.82 nm (less intense) and 336.82 nm (a broad and more intensity) were observed. On the other hand, a strong band with absorption maxima at 337.89 nm along with three more bands at 313.78 nm, 301.47 nm, and 283.77 nm were noticed in tri-DPM based truxene **18**. Noticeably, all three DPMs (**14**, **16**, and **18**) gave almost similar electronic spectra (Figure 2). Interestingly, even though we observed variations in the absorption spectra for thus prepared truxene-based molecules, but all the truxene derivatives displayed almost similar types of the emission spectra under identical conditions except the variations in the intensities of the bands. The bands observed for these compounds were found ranged from 350 to 508 nm, with a small shoulder in each case, which displays vibronic features (Figure 2). Exact values of the fluorescence maxima for these compounds are as follows: **12** (406.78, 428.93, and 457.04), **14** (406.42, 429.65, and 458.53), and **15** (406.42, 429.65, and 457.40), **16** (406.78, 430.48, and 457.40), **17** (406.18, 430.65, and 455.19), **18** (406.78, 430.42, and 457.40).

**Figure 3:**
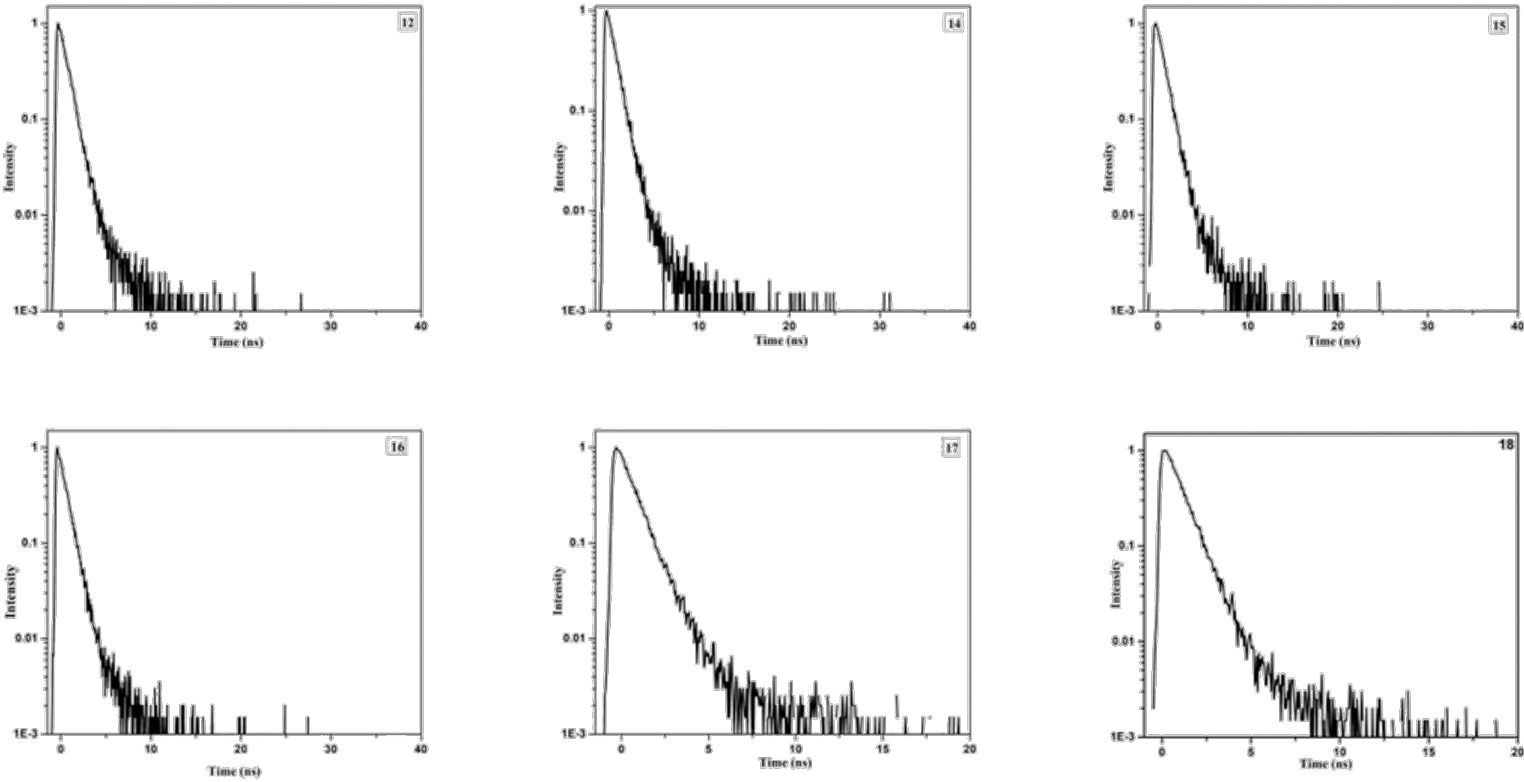
Time-resolved fluorescence lifetime.

Moreover, as can be seen from Figure 3 and Table 1, the time resolved fluorescence lifetime decays have also been investigated. Noticeably the fluorescence decays at around 457 nm were single exponential for all the compounds except for the compounds **16** and **17**.

**Table 1:** Fitting parameter of the fluorescence intensity decays of the truxene based DPMs.

Compound	λ_abs_ (nm)	λ_ems_ (nm)	τ_1_ (ns)	a_1_	a_2_	χ^2^

**12**	335	457	1.00	0.49	–	0.94
**14**	334	458	0.93	0.49	–	1.11
**15**	334	457	1.28	0.58	0.06	0.95
**16**	337	406	0.48	0.49	–	0.93
**17**	338	406	0.50	0.43	0.21	1.04
**18**	337	457	1.64	0.49	–	0.95

## Conclusion

In summary, novel truxene-based mono-, di- and tri-substituted dipyromethanes (DPMs) have successfully been synthesized. All the compounds were fully characterized and confirmed by means of the standard spectroscopic techniques like ^1^H NMR, ^13^C NMR, and mass spectral data. The preliminary UV–vis absorption as well as fluorescence emission spectral data for thus prepared truxene-based compounds were recorded in chloroform and compared as well. Additionally, time-resolved fluorescence lifetime decays were also measured for thus prepared compounds. The anion sensing/binding studies of these DPMs in addition to their formylated derivatives is under progress in our laboratory and will be published in due course. As mention above truxene and its congeners have shown a plethora of uses in diverse fields. To our best knowledge, their potential applications in the arena of supramolecular chemistry in general, sensing, molecular recognition and self-assembly in particular in has yet to be explored. Moreover, applications of the truxene derivatives in catalysis are also scared, and needs to be advanced in future research.

## Experimental

**General:** All the reagents/solvents were purchased from commercial suppliers (Sigma-Aldrich, Alfa Aesar, TCI, GLR innovation, Avera, Spectrochem and Across), and used without further purification. Solvents were dried according to standard reported procedures. Pyrrole was used after a fresh distillation. Analytical thin-layer chromatography (TLC) was performed on aluminium plates coated with silica gel by using a suitable mixture of EtOAc and petroleum ether for development purpose. Column chromatography was performed by using silica gel (100–200 mesh) with an appropriate mixture of EtOAc and petroleum ether. Characterization of all the compounds were accomplished using ^1^H, ^13^C NMR, and HRMS. See Supporting Information File 1 for the respective spectra. The “*’’ wherever present in NMRs spectra denotes solvent residual peaks. Chemical shifts are recorded in units of δ (ppm), and referenced with respect to the standard TMS. UV–vis data were recorded on a PerkinElmer (Lambda 365) UV–vis spectrophotometer in HPLC grade CHCl_3_.

**Synthesis of 10,15-dihydro-5*H*-diindeno[1,2-*a*:1',2'-*c*]fluorene (9):** The truxene scaffold **9** was prepared according to a literature report procedure [38]. l-Indanone (**8**, 6.8 g, 51.4 mmol) was added to a mixture of 60 mL acetic acid and 30 mL concentrated HCl, then the reaction mixture was stirred for 16 h at 100 °C. The creamy precipitate (4.7 g, 80%) was obtained after pouring the reaction mixture into crushed ice, washed with water, acetone and dichloromethane (DCM), then dried under vacuum to get the anticipated compound **9**.

**Synthesis of the 5,5,10,10,15,15-hexabutyl-10,15-dihydro-5*H*-diindeno[1,2-*a*:1',2'-*c*]fluorene (10)**: Pristine truxene **9** (4 g, 11.68 mmol), DMSO (35 mL), and *t*-BuOK (11.79 g, 105.12 mmol), were mixed in a two-necked round bottom (RB) flask (100 mL) under nitrogen atmosphere. The flask was cooled to 0 ºC, and stirred vigorously after that *n*-BuBr (11.30 mL, 105.12 mmol) was slowly added to the RB-flask, and the mixture was stirred for 24 h at room temperature (rt), until the reaction completed (TLC monitoring). Then the reaction mixture was quenched with water, and the crude product was extracted with ethyl acetate. The combined organic layers were washed with water (2 × 100 mL), dried over anhydrous Na_2_SO_4_. After evaporation of the solvent the residue was passed through a silica gel (SiO_2_) to give the final product as a white powder (7.5 g, 95%). The ^1^H NMR spectrum was perfectly matched with the previously reported one [38].

**General procedure for acetylation of truxene 10:** Compound **10** was dissolved in DCM (15 mL). This solution was gradually added to a AlCl_3_/acetyl chloride solution at 0 °C, which was prepared by dissolving AlCl_3_ in acetyl chloride (**11**) with different equivalents (2 equiv for **12**, 6 equiv for **15** and 9 equiv for **17**) at 0 °C under N_2_ atmosphere. The red reaction mixture was stirred for 30 min at 0 °C, and further stirred at room temperature for 1 to 6 hours. After the reaction completion (TLC monitoring), the mixture was poured gradually into crushed ice–cold water (≈150 mL), while stirring. The resulting mixture was then stirred at rt for 15 minutes. The aqueous solution was extracted with CH_2_Cl_2_, washed with saturated aq NaHCO_3_ solution, and dried over anhydrous Na_2_SO_4_. The solvent was removed under reduced pressure to afford the crude product which was then purified by silica gel column chromatography (as suitable mixture of ethyl acetate and petroleum ether) to give the anticipated products in **12** (85%), **15** (80%), and **17** (90%) yields.

**1-(5,5,10,10,15,15-Hexabutyl-10,15-dihydro-5*H*-diindeno[1,2-*a*:1',2'-*c*]fluoren-2-yl)ethan-1-one (12):** White solid; yield 85%; (0.9 g, starting from 1 g of **10**); *R*_f_ 0.50 (5% ethyl acetate/petroleum ether); mp 117–120 °C; ^1^H NMR (400 MHz, CDCl_3_) δ 8.47 (d, *J* = 8.3 Hz, 1H), 8.38 (d, *J* = 6.2 Hz, 2H), 8.08 (s, 1H), 8.02 (d, *J* = 8.2 Hz, 1H), 7.48 (d, *J* = 7.5 Hz, 2H), 7.40 (p, *J* = 7.1 Hz, 4H), 2.97 (m, 6H), 2.73 (s, 3H), 2.21–2.10 (m, 6H), 0.94–0.82 (m, 12H), 0.61–0.33 (m, 30H); ^13^C NMR (101 MHz, CDCl_3_) δ 198.17, 154.10, 153.58, 153.47, 146.43, 146.10, 146.00, 139.41, 137.24, 135.00, 127.27, 126.68, 126.66, 126.15, 124.81, 124.71, 124.34, 122.34, 122.29, 121.67, 55.74, 36.57, 26.80, 26.49, 22.83, 13.79; HRMS (*m*/*z*): [M + H]^+^ calcd for C_53_H_68_O, 721.5343; found, 721.5396.

**1,1'-(5,5,10,10,15,15-Hexabutyl-10,15-dihydro-5*H*-diindeno[1,2-*a*:1',2'-*c*]fluorene-2,7-diyl)bis(ethan-1-one) (15):** White solid; yield 80%; (0.9 g, starting from 1 g of **10**); *R*_f_ 0.60 (15% ethyl acetate/petroleum ether); mp 192–195 °C; ^1^H NMR (400 MHz, CDCl_3_) δ 8.42–8.39 (m, 2H), 8.32 (d, *J* = 7.2 Hz, 1H), 8.02 (d, *J* = 1.4 Hz, 2H), 8.00–7.93 (m, 2H), 7.45–7.41 (m, 1H), 7.38–7.32 (m, 2H), 3.01–2.81 (m, 6H), 2.66 (s, 6H), 2.19–2.00 (m, 6H), 0.81 (ddd, *J* = 15.7, 14.5, 7.3 Hz, 12H), 0.47–0.29 (m, 30H); ^13^C NMR (101 MHz, CDCl_3_) δ 198.17, 154.03, 153.92, 153.38, 147.37, 147.05, 144.97, 144.88, 139.56, 135.25, 127.43, 127.00, 126.35, 124.82, 124.48, 124.38, 122.37, 121.71, 55.99, 55.91, 55.85, 36.76, 36.58, 36.33, 26.81, 26.54, 26.49, 22.81, 22.75, 13.77; HRMS (*m*/*z*): [M + H]^+^ calcd for C_55_H_70_O_2_, 763.5449; found, 763.5454.

**1,1',1''-(5,5,10,10,15,15-Hexabutyl-10,15-dihydro-5*H*-diindeno[1,2-*a*:1',2'-*c*]fluorene-2,7,12-triyl)tris(ethan-1-one)** (**17**)**:** White solid; yield 90%; (0.9 g, starting from 1 g of **10**); *R*_f_ 0.65 (20% ethyl acetate/petroleum ether); mp 253–255 °C; ^1^H NMR (400 MHz, CDCl_3_) δ 8.48 (d, *J* = 8.4 Hz, 3H), 8.18–7.95 (m, 6H), 3.0–2.93 (m, Hz, 6H), 2.73 (s, 9H), 2.30–2.13 (m, 6H), 0.95–0.76 (m, 12H), 0.58–0.26 (m, 30H). The all data perfectly matched with the previously reported one [51].

**General procedure for the formation of truxene-based DPM derivatives 14, 16 and 18:** The truxene-based acetylated compounds **12**/**15**/**17**, were dissolved in freshly distilled pyrrole with different amounts (5 equiv for **14**, 10 equiv for **16,** and 15 equiv for **18**). Then trifluoroacetic acid (TFA, 0.1 equiv for **14**, 0.2 equiv for **16** and 0.3 equiv for **18**) was added to the reaction mixture, and the resulting mixture was stirred at 0 °C (**16**) or 0 °C to rt (**14**, **18**) for 8 h. After the reaction completion (TLC monitoring), excess of triethylamine (TEA) was added to quench the reaction mixture. After the removal of the unreacted pyrrole in vacuo (the temperature of the water bath and the pressure were set to 80 °C and 80 mbar, respectively), the dark brown residue was subjected directly to column chromatography over silica gel (20% EtOAc:hexane), to deliver the solid DPMs **14** (60%), **16** (71%), and **18** (80%).

**2,2'-(1-(5,5,10,10,15,15-Hexabutyl-10,15-dihydro-5*H*-diindeno[1,2-*a*:1',2'-*c*]fluoren-2-yl)ethane-1,1-diyl)bis(1*H*-pyrrole) (14):** Gray solid; yield 60% (278 mg, starting from 400 mg of **12**); *R*_f_ 0.70 (20% ethyl acetate/petroleum ether); mp 120–123 °C; ^1^H NMR (400 MHz, CDCl_3_) δ 8.34 (dd, *J* = 19.2, 7.3 Hz, 2H), 8.21 (d, *J* = 8.4 Hz, 1H), 7.88 (s, 2H), 7.50–7.42 (m, 2H), 7.42–7.31 (m, 5H), 6.96 (dd, *J* = 8.3, 1.8 Hz, 1H), 6.73 (dd, *J* = 4.2, 2.6 Hz, 2H), 6.22 (dd, *J* = 6.0, 2.8 Hz, 2H), 6.10–5.95 (m, 2H), 3.07–2.79 (m, 6H), 2.16 (s, 3H), 2.04 (m, 6H), 0.92–0.83 (m, 12H), 0.57–0.38 (m, 30H); ^13^C NMR (101 MHz, CDCl_3_) δ 153.57, 145.53, 144.33, 140.15, 138.42, 137.84, 126.35, 125.98, 124.70, 122.27, 121.52, 116.88, 108.26, 106.37, 96.62, 55.52, 55.45, 44.87, 36.62, 36.37, 26.57, 26.49, 22.84, 22.79, 13.83, 13.80; HRMS (*m*/*z*): [M + H]^+^ calcd for C_61_H_76_N_2_, 837.6081; found, 837.6135.

**2,2',2'',2'''-((5,5,10,10,15,15-Hexabutyl-10,15-dihydro-5*H*-diindeno[1,2-*a*:1',2'-*c*]fluorene-2,7-diyl)bis(ethane-1,1,1-triyl))tetrakis(1*H*-pyrrole) (16):** Brown solid; yield 71% (185 mg, starting from 200 mg of **15**); *R*_f_ 0.43 (20% ethyl acetate/petroleum ether); mp 110–112 °C; ^1^H NMR (400 MHz, CDCl_3_) δ 8.31 (d, *J* = 7.5 Hz, 1H), 8.18 (dd, *J* = 17.9, 8.4 Hz, 2H), 7.88 (s, 4H), 7.48–7.29 (m, 5H), 6.94 (dd, *J* = 8.3, 1.7 Hz, 2H), 6.77–6.67 (m, 4H), 6.22 (dd, *J* = 5.8, 2.8 Hz, 4H), 6.04 (s, 4H), 2.97–2.77 (m, 6H), 2.15 (s, 6H), 2.06–1.90 (m, 6H), 0.93–0.83 (m, 12H), 0.54–0.43 (m, 30H); ^13^C NMR (101 MHz, CDCl_3_) δ 153.53, 153.46, 145.28, 145.09, 144.90, 140.31, 139.03, 138.00, 137.83, 126.36, 125.99, 125.03, 124.31, 124.26, 122.28, 121.65, 116.90, 108.25, 106.38, 55.51, 55.44, 44.87, 36.53, 36.37, 36.29, 26.60, 26.56, 26.51, 22.84, 22.81, 22.79, 13.88, 13.85, 13.83; HRMS (*m*/*z*): [M + H]^+^ calcd for C_71_H_86_N_4_, 995.6925; found, 995.6991.

**2,2',2'',2''',2'''',2'''''-((5,5,10,10,15,15-Hexabutyl-10,15-dihydro-5*H*-diindeno[1,2-*a*:1',2'-*c*]fluorene-2,7,12-triyl)tris(ethane-1,1,1-triyl))hexakis(1*H*-pyrrole) (18):** Light orange solid; yield 80% (458 mg, starting from 400 mg of **17**); *R*_f_ 0.44 (20% ethyl acetate/petroleum ether); mp 160–162 °C; ^1^H NMR (400 MHz, CDCl_3_) δ 8.15 (d, *J* = 8 Hz, 3H), 7.89 (s, 6H), 7.34 (s, 3H), 6.96 (s, 3H), 6.74 (s, 6H), 6.24 (s, 6H), 6.06 (s, 6H) 2.84 (s, 6H), 2.18 (s, 9H), 1.97 (s, 6H), 0.89 (s, 12H), 0.50 (m, 30H); ^13^C NMR (101 MHz, CDCl_3_) δ 153.43, 145.30, 145.06, 138.97, 137.97, 137.82, 125.03, 124.26, 121.67, 116.92, 108.24, 106.38, 55.42, 44.86, 36.28, 29.01, 26.59, 22.80, 13.88; HRMS (*m*/*z*): [M + 2H]^+^ calcd for C_81_H_96_N_6_, 1154.7842; found, 1154.7771.

## Supporting Information

File 1 ^1^H NMR, ^13^C NMR and HRMS spectra of all the synthesized compounds.

## Data Availability

All data that supports the findings of this study is available in the published article and/or the supporting information to this article.
