# Supplementary material for: Novel truxene-based dipyrromethanes (DPMs): synthesis, spectroscopic characterization and photophysical properties
Source: Beilstein J Org Chem. 2024 Aug 29;20:2163–70. doi: 10.3762/bjoc.20.186 (PMC11368050; doi:10.3762/bjoc.20.186)

BEILSTEIN JOURNAL OF ORGANIC CHEMISTRY

# Supporting Information

for

# Novel truxene-based dipyrromethanes (DPMs): synthesis, spectroscopic characterization and photophysical properties

Shakeel Alvi and Rashid Ali

*Beilstein J. Org. Chem.* **2024,** *20,* 2163–2170. doi:10.3762/bjoc.20.186

## $^{1}H$ NMR, $^{13}C$ NMR and HRMS spectra of all the synthesized compounds

License and Terms: This is a supporting information file under the terms of the Creative Commons Attribution License (https://creativecommons.org/licenses/by/4.0). Please note that the reuse, redistribution and reproduction in particular requires that the author(s) and source are credited and that individual graphics may be subject to special legal provisions.
The license is subject to the *Beilstein Journal of Organic Chemistry* terms and conditions: (https://www.beilstein-journals.org/bjoc/terms)

# Table of contents


| | |
|---|---|
| $^{1}H$ NMR and $^{13}C$ NMR spectrum of compound **12** | S2 |
| HRMS spectrum of compound **12** | S3 |
| $^{1}H$ NMR spectrum of compound **14** | S3 |
| $^{13}C$ NMR and HRMS spectrum of compound **14** | S4 |
| $^{1}H$ NMR and 13C NMR spectrum of compound **15** | S5 |
| HRMS spectrum of compound **15** | S6 |
| $^{1}H$ NMR spectrum of compound **16** | S6 |
| $^{13}C$ NMR and HRMS spectrum of compound **16** | S7 |
| $^{1}H$ NMR spectrum of compound **17** | S8 |
| $^{1}H$ NMR spectrum of compound **18** | S8 |
| $^{13}C$ NMR and HRMS spectrum of compound **18** | S9 |

$^{1}$H NMR spectrum of compound **12**

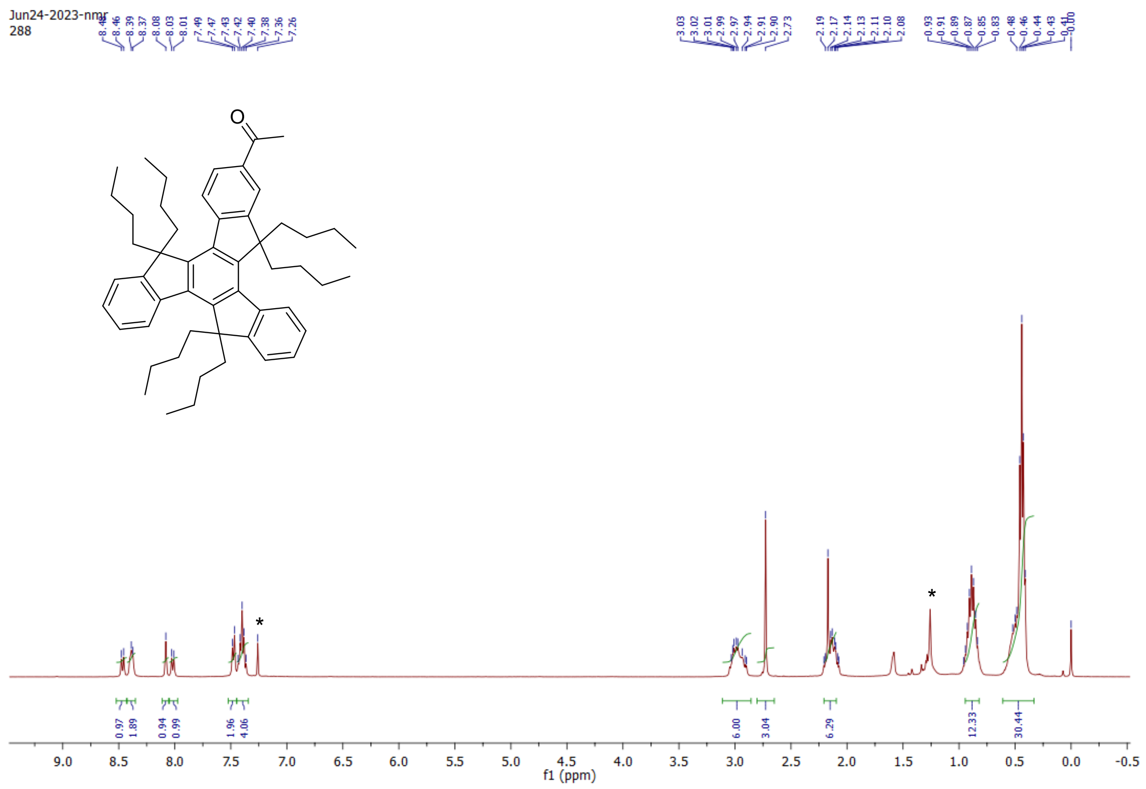


$^{13}$C NMR spectrum of compound **12**

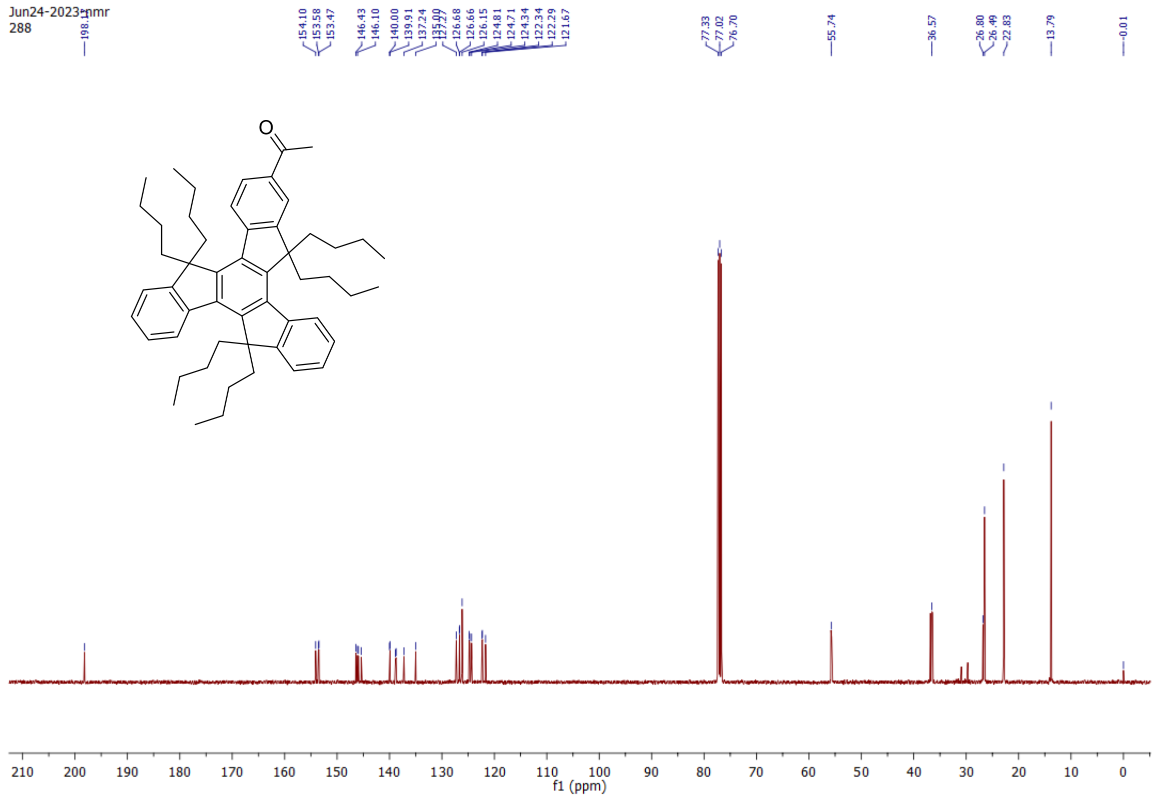

HRMS spectrum of compound **12**

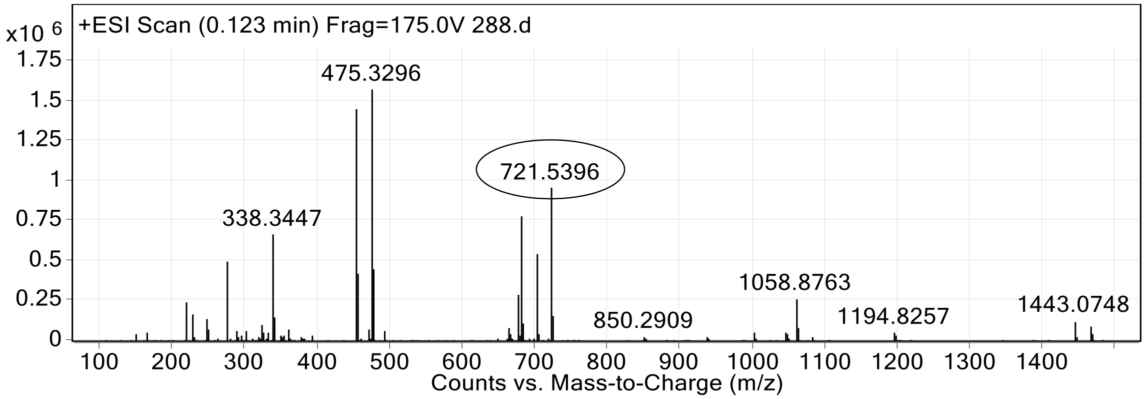


$^{1}$H NMR spectrum of compound **14**

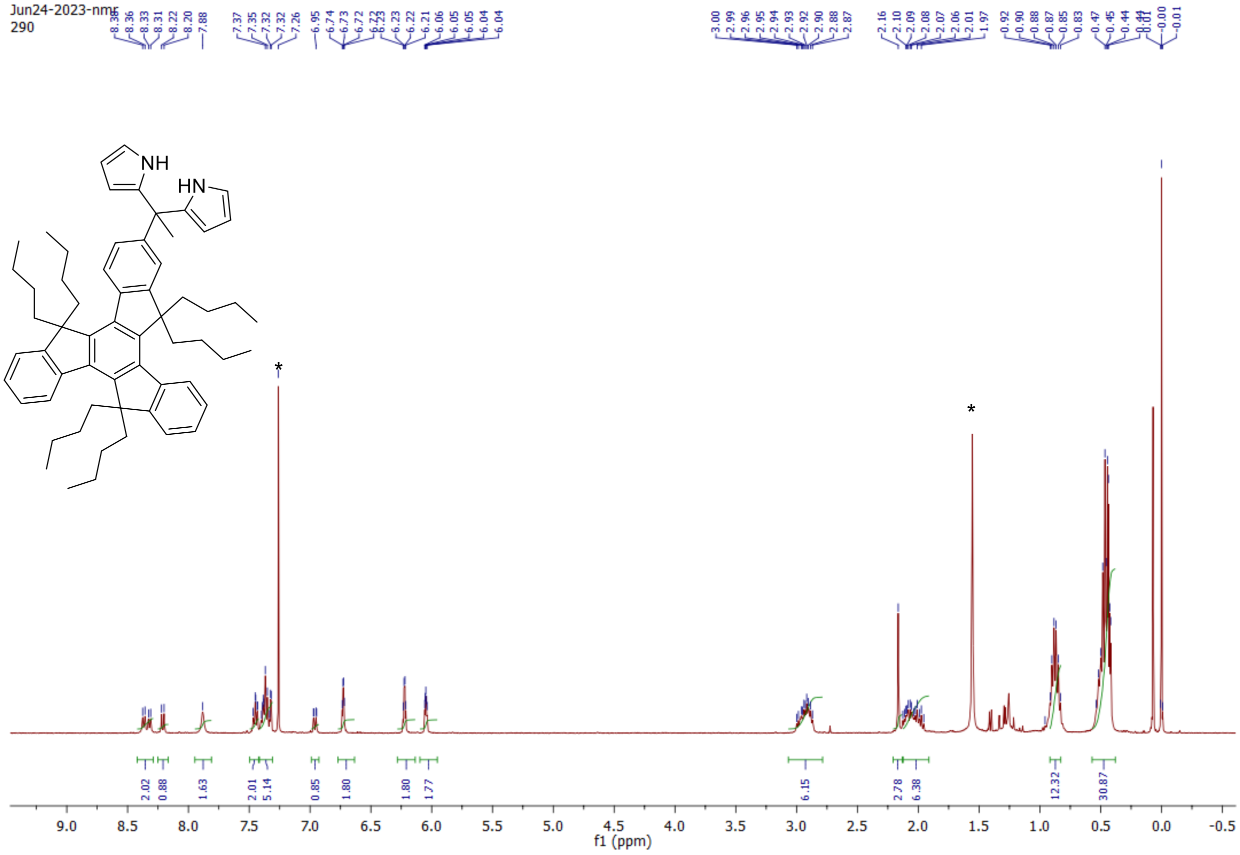

$^{13}C$ NMR spectrum of compound **14**

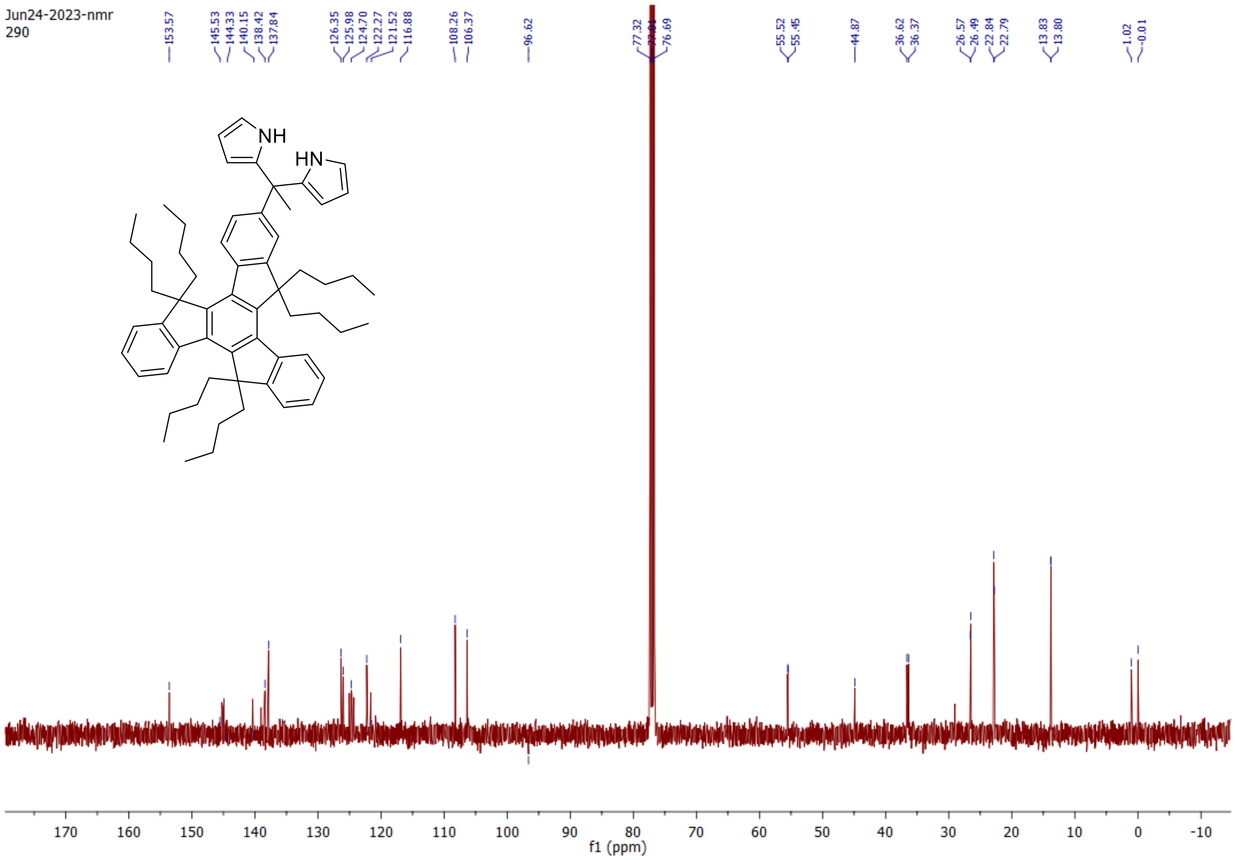


HRMS spectrum of compound **14**

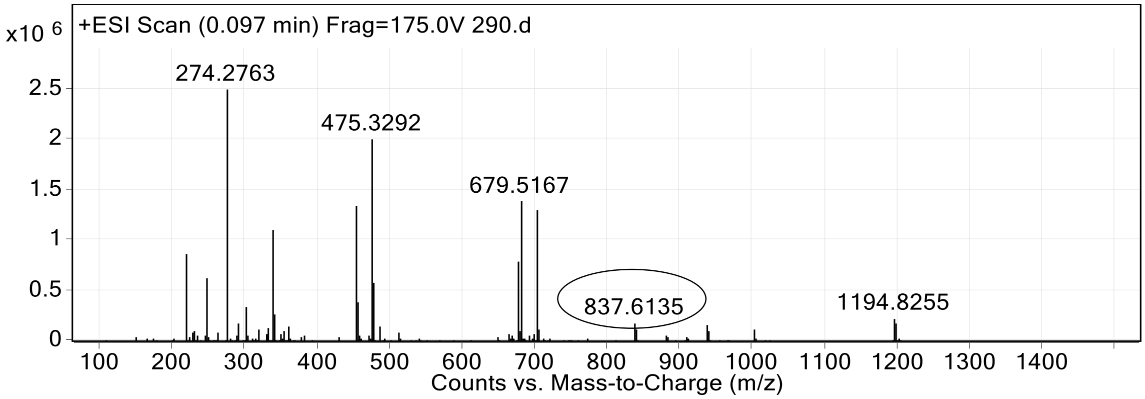

$^{1}$H NMR spectrum of compound **15**

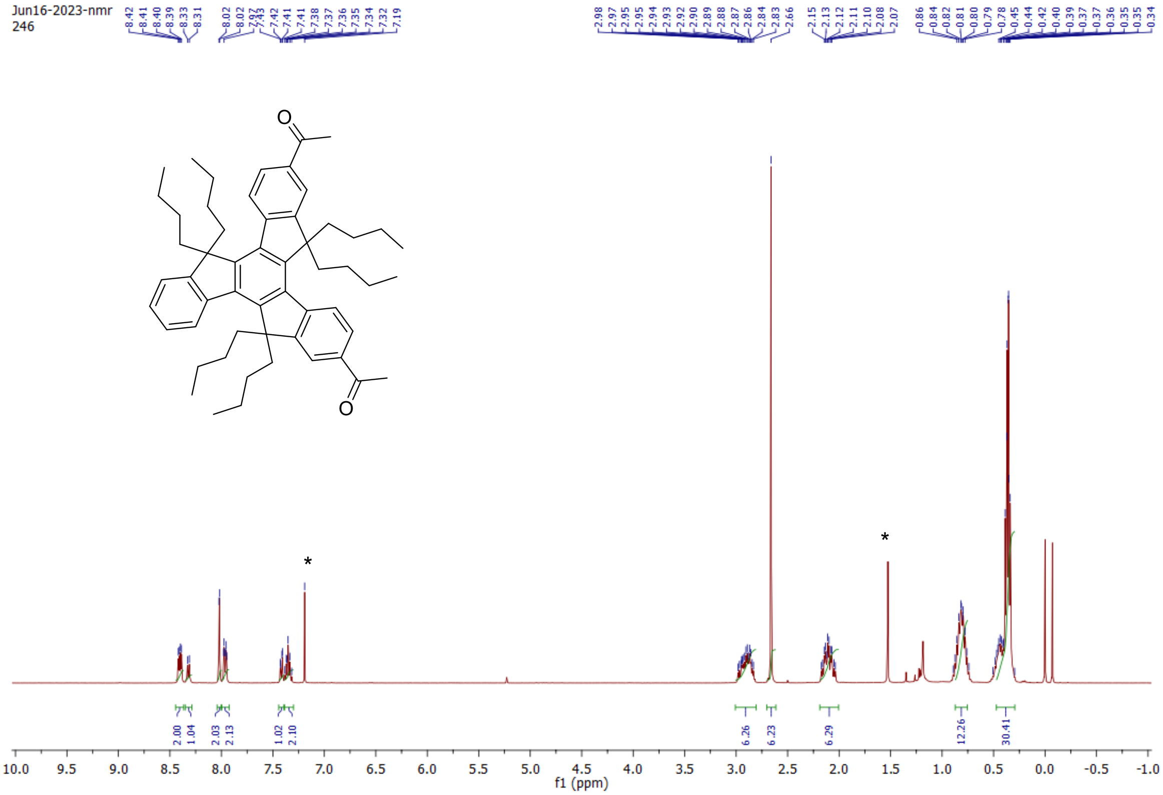


$^{13}$C NMR spectrum of compound **15**

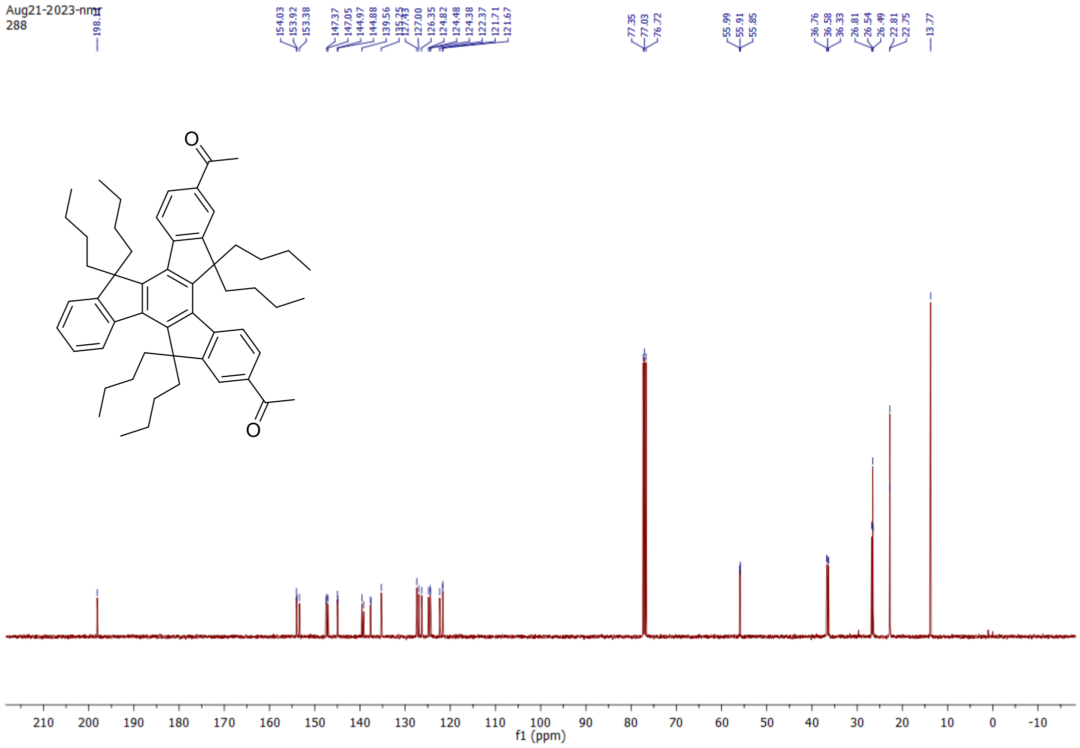

HRMS spectrum of compound **15**

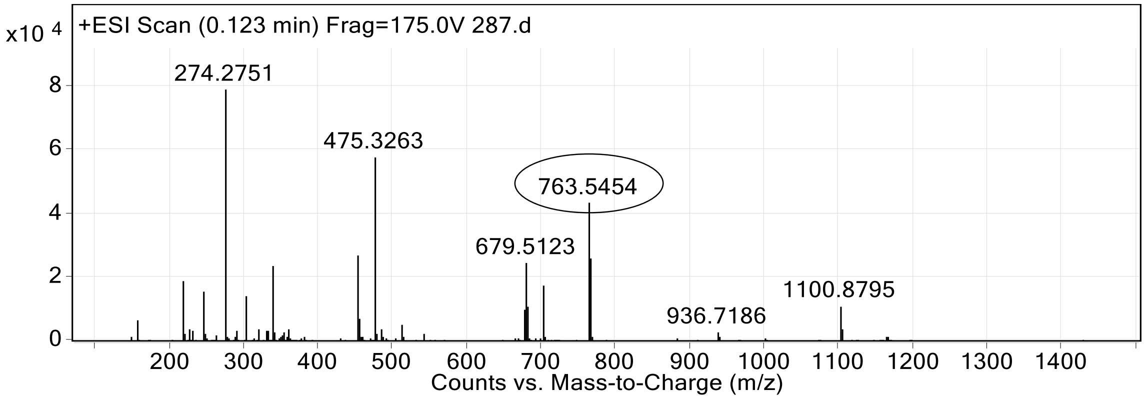


$^{1}$H NMR spectrum of compound **16**

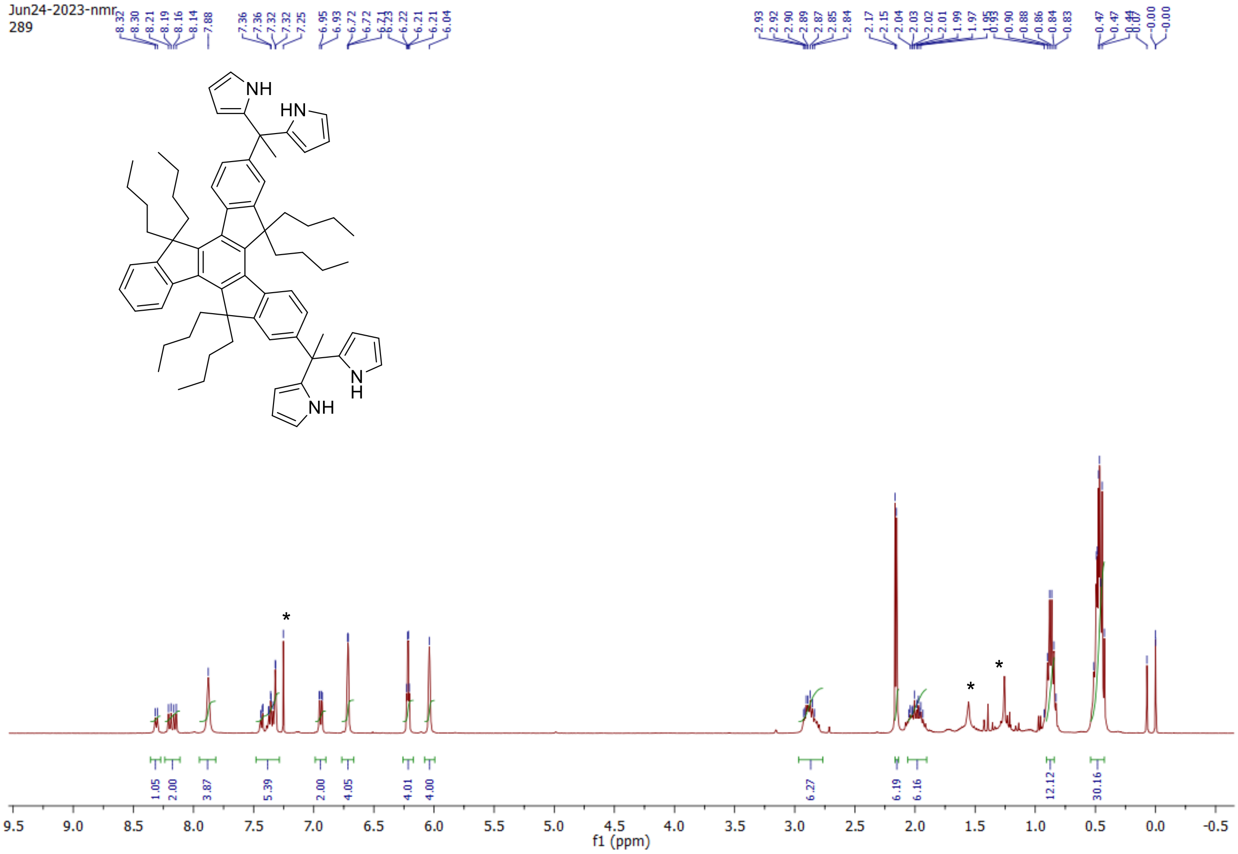

$^{13}$C NMR spectrum of compound **16**

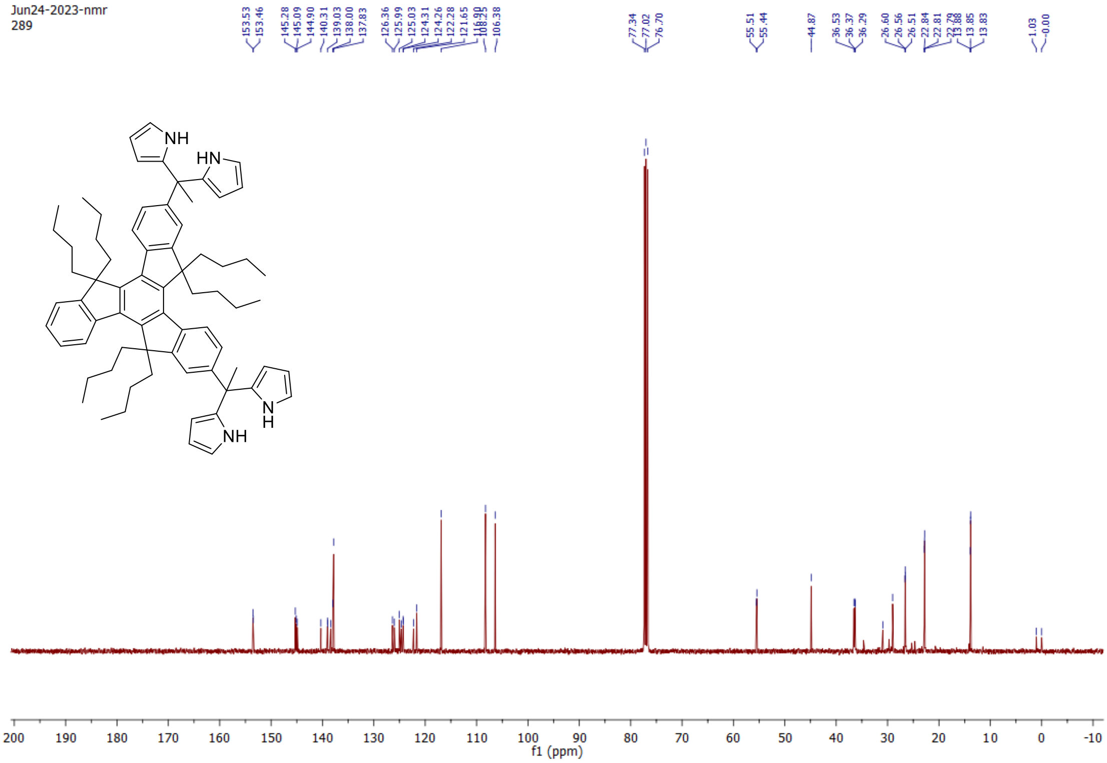


HRMS spectrum of compound **16**

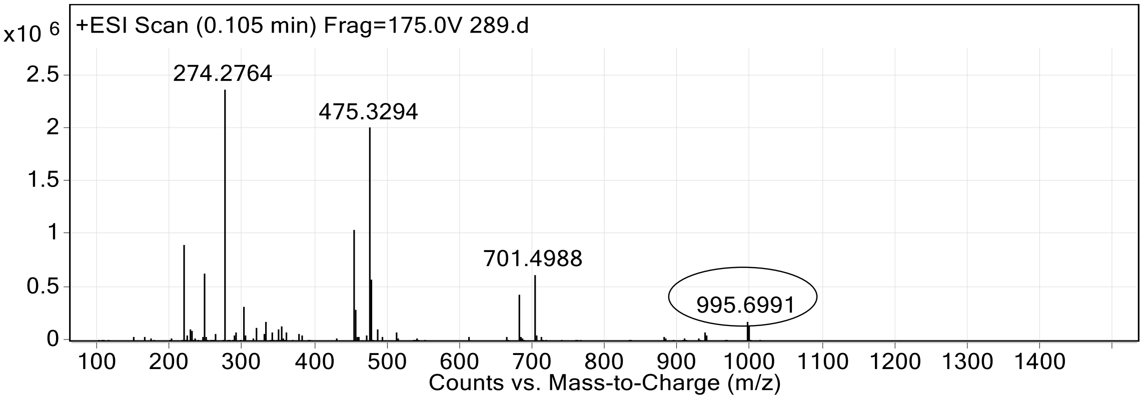

$^{1}$H NMR spectrum of compound **17**

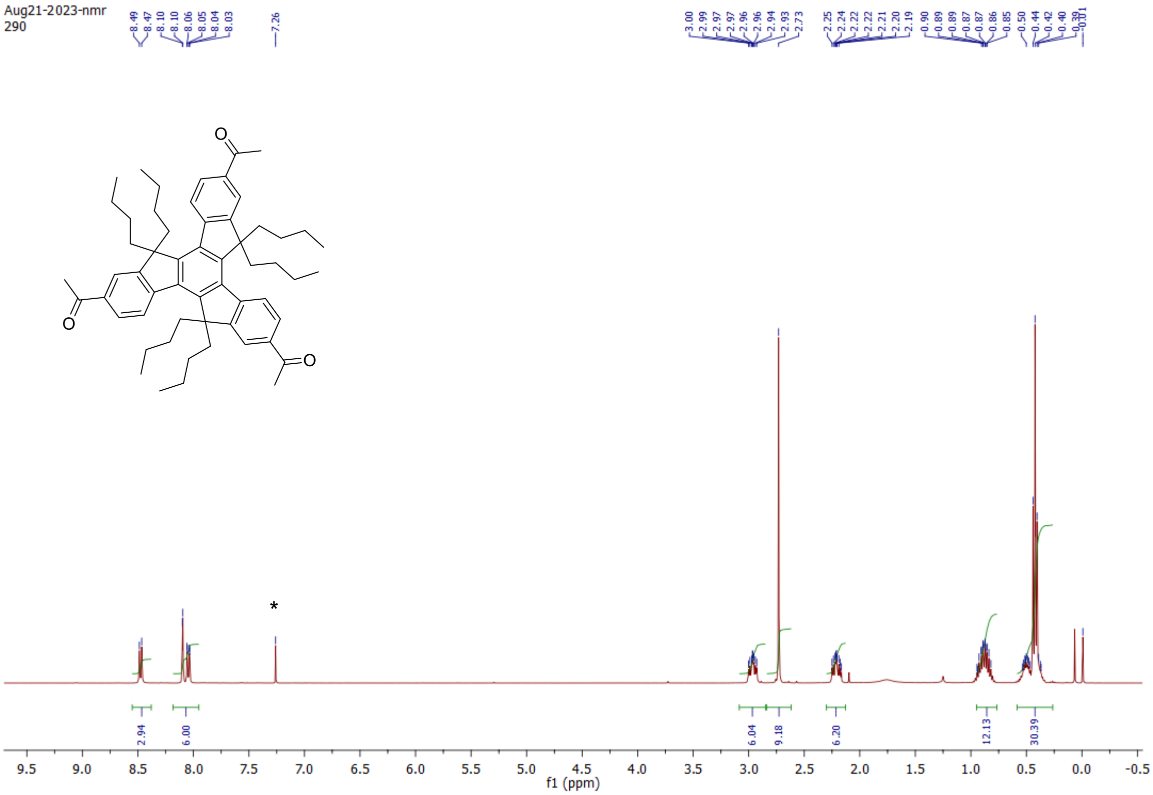


$^{1}$H NMR spectrum of compound **18**

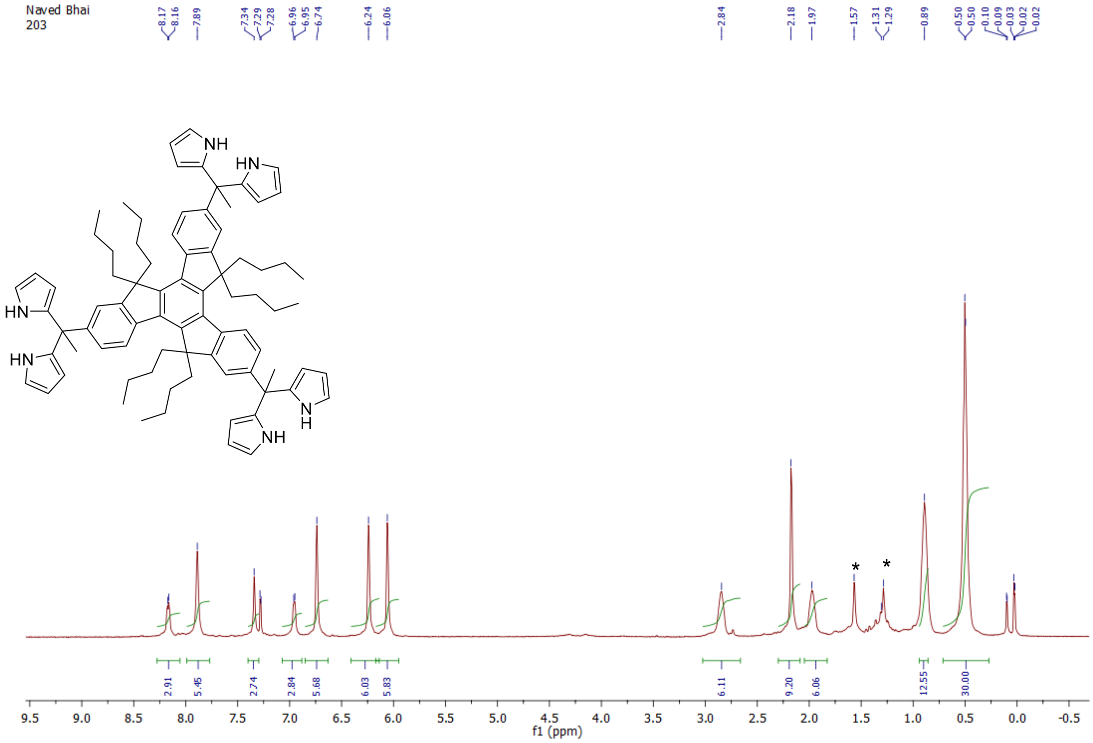

$^{13}$C NMR spectrum of compound **18**

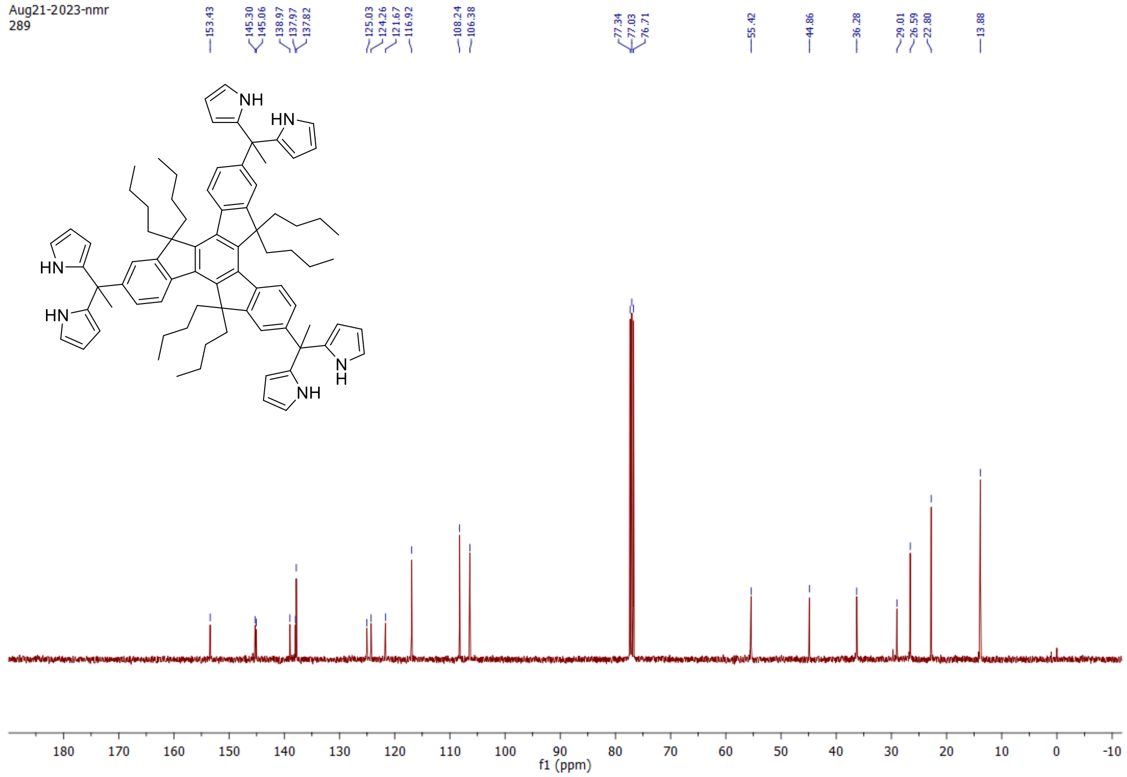


HRMS spectrum of compound **18**

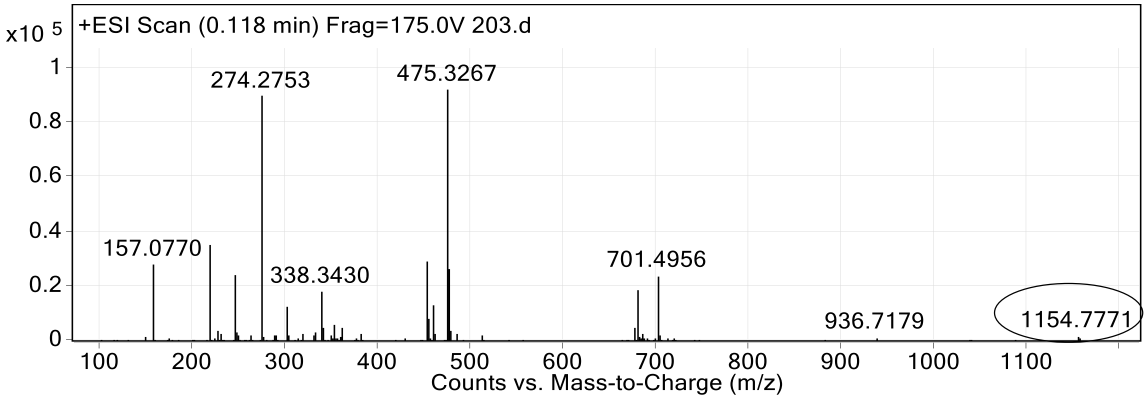

Supplement: File 1 — 1H NMR, 13C NMR and HRMS spectra of all the synthesized compounds. [file Beilstein_J_Org_Chem-20-2163-s001.pdf]
